# Food insecurity indicators of 14 OECD countries in a health economics aspect: A comparative analysis

**DOI:** 10.3389/fpubh.2023.1122331

**Published:** 2023-04-06

**Authors:** Salim Yılmaz, Ahmet Murat Günal

**Affiliations:** ^1^Department of Health Management, Faculty of Health Sciences, Istanbul Arel University, Istanbul, Türkiye; ^2^Department of Nutrition and Dietetics, Faculty of Health Sciences, Istanbul Okan University, Istanbul, Türkiye

**Keywords:** food insecurity, OECD countries, health policy, health spending, health economics

## Abstract

**Introduction:**

Food insecurity is a critical issue that refers to a lack of access to adequate food to support a healthy and active lifestyle. This problem has wide-reaching effects and can negatively impact health, education, and overall well-being. Addressing food insecurity requires a multifaceted approach that involves the efforts of governments, organizations, and individuals to ensure access to a balanced and nutritious diet for all.

**Methods:**

The aim of this study is to shed light on macro-level models and evaluate food insecurity risk in international comparisons. We considered six criteria to evaluate food insecurity risk in terms of health expenditure, gross domestic product (GDP) *per capita*, and GDP growth rate among 14 Organisation for Economic Co-operation and Development (OECD) countries. We developed a modeling approach in three stages to compare food insecurity risk and discussed the reasons for the rankings of the countries based on the model results.

**Results:**

According to our findings, the United States has the lowest food insecurity risk, while Colombia has the highest. The results suggest that economic factors, such as GDP per capita and GDP growth rate, play a significant role in food insecurity risk. The study highlights the importance of addressing economic disparities and promoting economic growth to reduce food insecurity.

**Discussion:**

This study provides insights into the relationship between food insecurity and economic factors, indicating that addressing economic disparities and promoting economic growth can reduce food insecurity. Future research using similar models to link economic outcomes with important health components such as nutrition and physical activity could provide a foundation for policy development.

## 1. Introduction

Climate crisis, the COVID-19 pandemic, increasing inflation, fear of recession, and the ongoing war between Ukraine and Russia, two essential grain importers, the world is threatened once more with a not-so-foreign term, food insecurity.

The definition of food security is agreed upon in World Food Summit 1996 as “When all people, at all times, have physical and economic access to sufficient safe and nutritious food that meets their dietary needs and food preferences for an active and healthy life.” Thus, food insecurity refers to a lack of access to enough food to support a healthy and active lifestyle (1). It is a complex issue that can have a variety of causes and effects. Some of the foremost causes of food insecurity include poverty, natural disasters, conflict, and inadequate infrastructure for food distribution. These factors can make it difficult for people to access enough food to support a healthy and active lifestyle. Food insecure people may have difficulty getting enough to eat, may not have access to a diverse and nutritious diet, or may have to resort to eating less healthy food to make ends meet (2).

The effects of food insecurity are wide-reaching and can have negative impacts on health, education, and overall well-being. For example, food insecurity can lead to malnutrition, which can have serious health consequences, particularly for children. Malnutrition can cause a range of health problems, including stunted growth, weakened immune systems, and increased susceptibility to illness. When children are not getting enough to eat, they may be less able to focus and learn in school. This can lead to reduced school attendance and lower educational achievement. Additionally, food insecurity can lead to increased stress and anxiety levels, which can negatively impact mental health (3). Food insecurity can also have broader societal impacts, such as increased crime rates and reduced economic productivity (4).

Overall, food insecurity is a serious problem affecting millions of people worldwide and requires a multifaceted approach to address. Governments, organizations, and individuals need to work together to address the root causes of food insecurity and to ensure that everyone has access to enough food to support a healthy and active lifestyle.

## 2. Literature review

Wars, pandemics, increasing population, and income inequalities are increasingly exacerbating the threat of food insecurity. Furthermore, even before the pandemic, global efforts to control the rising food insecurity by 2030 had not been successful (5). Hunger, poverty, and food insecurity are closely linked to malnutrition, and research has mapped longitudinal changes in gross domestic product (GDP) *per capita* (6–8). Between 1990 and 2010, indicators such as daily energy consumption and meat consumption increased in East Asian and Pacific countries, with an average growth of 2% (6). Warr (9) stated that the GDP growth rate reduces food insecurity and that GDP *per capita* is even more effective in doing so. Beckman et al. (10) found that in the Organisation for Economic Co-operation and Development (OECD) countries, the 7.2% decrease in GDP *per capita* during the COVID-19 period caused a 27.8% increase in the number of people affected by food insecurity and a 9% decrease in the income of crop producers. Moreover, responses to crises also pose an additional threat to groups experiencing food insecurity. For example, in a study conducted in Korea comparing the food insecurity situation before the pandemic in 2019 and after the pandemic in 2020, the vitamin C intake and fruit consumption of individuals in insecure situations remained significantly lower compared to the changes observed in the group with secure food status (11).

There is a correlation between the increase in food insecurity worldwide and the increase in chronic diseases (12). Moreover, studies show that food insecurity may have a two-way relationship with cardiovascular diseases (13, 14). Food insecurity plays a significant role in chronic diseases that put a strain on the health system financially, increasing health expenditures. As countries strive to increase access to comprehensive health services, they are also forced to increase the share of health expenditures in GDP (15). In such a situation, GDP cannot show strong growth in the short term in the face of increasing diseases and population, and the risk of food insecurity is also increasing worldwide (6). In addition to the inevitable increase in health expenditures with an aging population, it is discussed that the risk of food insecurity may put the health system in a more difficult situation in the coming years (16). On the other hand, governments are inclined to determine their policies on healthcare systems according to the expectations of the public, despite all their drawbacks. However, misinformation and various speculative discourses may still inadequately affect public preferences, despite government support. Thus, various socio-demographic factors may indirectly become determinants of the healthcare system and policies. For instance, during the COVID-19 pandemic, it has been shown that age, gender, educational level, and economic prosperity may have an impact on vaccination behavior in terms of vaccine hesitancy and skepticism (17). In addition, it is reported that gender-based opportunity inequalities, economic income inequalities, and gaping differences in educational levels exacerbate this situation (18). Retrospectively, today’s world is paying the price of not being able to solve the food insecurity problems of previous years, and it is foreseeable that it will face rising risks in the coming years.

In recent years, political developments have also left indirect evaluations based only on health spending, GDP, and GDP growth rate insufficient. The grain crisis caused by Russia’s blockade has reduced the global food supply (19). This situation may bring to the fore the possibility that countries with high production capacity for crops may be less affected by political developments that increase the risk of food insecurity. As a proactive approach to these developments increases the focus on sustainable and resilient food systems, certainly producer protection and producer support have also increased their weight in the dimensions that make up food insecurity (20). While efforts are being made to develop collaborations and protocols for greater transnational integration regarding agriculture and food production and transportation, on the other hand, the insufficient provision of support and protection for agricultural producers continues to pose a significant risk of food insecurity (21). Latino et al. (22) have associated models that try to solve problems such as food supply chains and waste reduction with countries that prioritize the role of local producers in consumption. Accordingly, the low *per capita* production and consumption of local crops in countries that are dependent on imports increases food insecurity risk. In the OECD data, ton-based information of countries in terms of wheat, maize, rice, and soybean with crop production, and the importance of crop production is related to harvested areas, returns per hectare (yields), and quantities produced are shared (23). Despite the fact that food insecurity resulting from insufficient crop production is of utmost importance, it creates both causal and consequential effects. This is due to factors such as decreasing water resources as a result of increasing urbanization and population density, increasing demand for local food transportation and foreign food imports, increasing waste, acquisition of some nutrition habits specific to large cities that threaten health, as well as the loss of agricultural land and required workforce in agriculture due to poverty caused by unemployment (24). Producer protection is directly related to crop production and is defined as the ratio between the average price received by producers (measured at the farm gate), including net payments per unit of current output, and the border price (measured at the farm gate) (25). Meanwhile, according to OECD data sharing, producer support is defined as a subgroup of the agricultural support indicator. Agricultural support is the annual monetary value of gross transfers to agriculture from consumers and taxpayers arising from government policies that support agriculture, regardless of their objectives and economic impacts (26). Another important OECD indicator in terms of crop production and consumption is meat consumption. Meat consumption is related to living standards, diet, livestock production, and consumer prices, as well as macroeconomic uncertainty and shocks to GDP, and OECD data includes beef and veal, pig, poultry, and sheep per kilogram *per capita* (27). Approximately 200 million people in India suffer from inadequate nutrition and struggle with consuming meat and obtaining protein (28). Consumption of meat is an ecologically controversial issue in terms of meat production, based on the increase in CO2 emissions. However, the correlation between the high level of GDP and excessive use of vehicles complicates the issue. Nevertheless, it is a known fact that population growth is the biggest threat in this regard and is closely related to the risk of food insecurity. In this context, the ability to consume meat can be considered a critical variable for food insecurity (29). Between 2017 and 2021, household spending accounted for 60.5% of India’s GDP and contributed to poverty and food insecurity among the Indian people (30). Although poverty appears to be less on average for OECD countries, food insecurity still exists with differences. In some developed countries, the excessive share of household spending in GDP increases food insecurity and contributes to income inequality (31). According to the OECD, household spending refers to the amount of money that resident households spend on final consumption to meet their daily needs, such as food, clothing, housing (rent), energy, transportation, durable goods (including cars), health care costs, leisure, and miscellaneous services (32). Increasing food inflation in recent years directly threatens food insecurity. Households struggling with poverty face financial constraints that lead to inadequate food intake. Food inflation is typically measured using the consumer price index (CPI), which is the change in the prices of a basket of goods and services commonly purchased by households (33). The ongoing war between two major agricultural powers, Ukraine, and Russia, may lead to significant food disruptions in Middle Eastern and North African countries that follow an import-based model for agricultural products, according to Ben Hassen and El Bilali (20). All of this shows that indicators such as producer protection and support, crop production, and meat consumption are in a two-way relationship with each other in terms of risk assessment for food insecurity and can destructively create economic problems, especially for the poor, and increase the need for health spending.

The inadequate data on the determinants of food insecurity risk, the difficulty of estimating, and the fact that it is a multidimensional concept makes it difficult to determine. However, comparative analyses under certain explanatory variables will serve as a resource for policy determination by discussing the reasons for the countries’ positions. Urbanization, limited access to water resources, decreasing workforce in agriculture and animal husbandry, rapid decline in the young population, rapidly increasing population, technological access level in production and logistics, climate change, and many other factors are among the other elements that cause food insecurity. In addition, health outcomes that approach food insecurity and create consequential effects, such as difficulties in accessing health services, trained health workforce, habits, and addictions, certainly represent other causes from a health economics perspective. This study sets the research limitations on six food insecurity variables and three health economics area variables related to health economics and food insecurity areas that can be evaluated within a very broad framework and compares countries based on these variables. Thus, determining the situations of food insecurity risks of countries compared to each other in the framework of health economics is the basic hypothesis. Additionally, determining the importance coefficients of which variables according to health economics outputs, revealing the descriptive data of countries regarding these variables, and evaluating the findings of all these situations are among the objectives of the study.

## 3. Materials and methods

Our study aims to use data from 14 OECD countries to determine the weights of food insecurity dimensions based on risk, using health spending *per capita*, GDP *per capita*, and GDP growth rate, and to rank the countries according to these weights. We also evaluated the 14 OECD countries according to six criteria of food insecurity that we have defined based on this weighted approach.

### 3.1. Research data

We have used OECD data in this study to ensure that all the data comes from the same source. We have chosen six widely accepted indicators of food insecurity as our criteria for 14 OECD countries for the year 2020. These are total (beef and veal; pork; poultry; sheep) meat consumption (kg *per capita*), total (wheat, maize, rice, soybean) crop production (tons per 1 m people), producer protection (total ratio), producer support (% of gross farm receipts), food inflation (annual growth rate), household spending (% of GDP). Also, we determined GDP *per capita*, health spending, and GDP growth rate as three indirect variables; that interact with each other and the other criteria in terms of both long-term outcome and cause. We are using this data because it is the most recent data available for the year 2020 in the OECD database for all 14 countries in terms of the relevant variables for the study. These ensure our data is up-to-date and allow us to accurately analyze and compare the food insecurity dimensions across the 14 countries.

### 3.2. Research model

We completed the research model by planning it in three stages. In the first stage, we performed a factor analysis using the principal component matrix method on health spending *per capita*, GDP *per capita*, and GDP annual growth rate variables. We scored the factor using the regression scores method for the observations, creating a single factor under the influence of the three variables. We coded the factor name as Factor_1.

In the second stage, we determined the criterion weights by identifying the distance of the six variables from Factor_1. We incorporated this distance into the model using the Multidimensional Scaling Method (MDS) and calculated it using the Euclidean distance method. We calculated the distance ratios of the six variables from Factor_1 and subtracted them from 1 for ranking the results of the ratio matrix from the lowest risk of food insecurity. Then, to determine the criterion weights, we compared them to the total score. These ratios formed the criterion weights.

**Figure 1:**
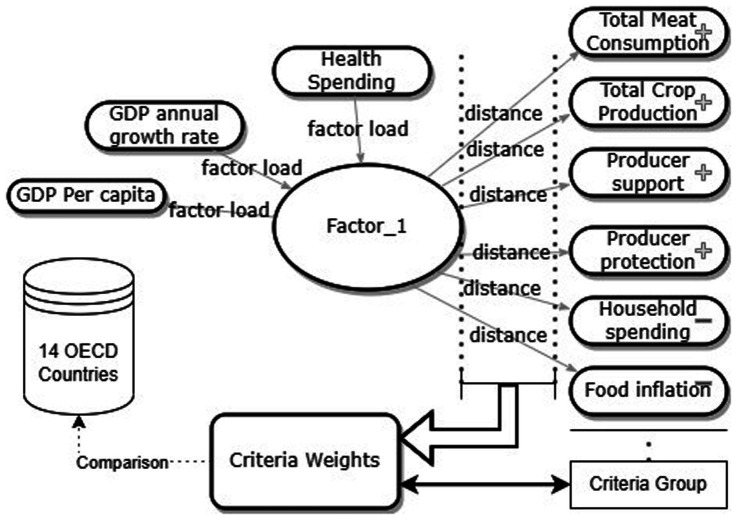
Research model.

In the last step, we used the Technique for Order of Preference by Similarity to Ideal Solution (TOPSIS) method to determine the country with the lowest risk of food insecurity within a set of 6 variables and to rank them from lowest to highest risk. Another reason we used this method is that it considers variables that have positive and negative effects. In the model, we determined that total meat consumption, crop production, producer protection, and producer support are positive, while food inflation and household spending are negatively effective. The research model is shown in Figure 1.

### 3.3. Limitations of study

Food insecurity is affected by many interrelated economic data. In our research, we aimed to use as recent data as possible from the OECD, to compare as many OECD countries as possible, and to include as many criteria as possible that are believed to affect food insecurity and are logically related. Therefore, using data from 2020, including 14 OECD countries, and comparing them based on six criteria were the most significant limitations of the study.

### 3.4. Statistical analysis

We used IBM SPSS 22.0 and MS Excel 16 programs. We interpreted the factor analysis at a 95% confidence level. In the solution-oriented analysis section, analysis was performed using three different and interconnected stages of statistical methods. The first of these is Factor Analysis, which aims to reduce numerous Nutrition Insecurity variables to a single variable and determine the weights of the converging other variables. The variables GDP *per capita*, GDP annual rate, and health expenditure ($) were combined under a single factor. Then, the values of Factor_1 were determined for each country using regression outputs. The generalized method of moments (GMM) was used to obtain the regression outputs in a similar way as applied to cross-sectional data. However, the GMM method is a technique that allows the use of lagged levels of regressors (explanatory factors) as instruments to address the probable link between the lagged regress and the error term, as well as the endogeneity of explanatory factors (34). In our study, cross-sectional data is evaluated from a single time period. Cross-sectional data represents the analysis of n variables related to different variables at a single time, rather than time-series data (35).

In the second stage, Multi-Dimensional Scaling (MDS), which is a multi-factor evaluation technique in cross-sectional data, was used (36). This method is a multivariate technique that can process metric data on an ordinal or nominal scale and measure distances (Euclidean Distance) to the point where the reduced data converge (37). In the MDS analysis, we created priorities using the PROXCAL method. We determined the distance matrix based on variables using *Z* scoring transformation. We checked the model for minimum stress and S-stress values using congruence indices. We determined the proportional weights of the distances and accepted them as criterion weights.

In the final step, we compared the countries using the Technique for Order Preference by Similarity to Ideal Solution (TOPSIS) method based on the criterion weights we determined. In the TOPSIS method, determining the decision option that is closest to the positive ideal solution and farthest from the negative ideal solution through matrices is targeted by a multicriteria approach (38). To make the comparison, we created a benefit and cost matrix from the weighted normalized matrix we determined. Using the benefit and cost matrix, we determined the positive and negative ideal solution values for the observations. We ranked the countries from the one with the lowest risk of food insecurity to the one with the highest by calculating the closeness coefficient of the results we obtained.

## 4. Results

In Table 1, the data of the total meat consumption (kg *per capita*) and crop production (tons per 1 m people) and their subunits, population, producer protection (total ratio), producer support (% of gross farm receipts), household spending (% of GDP), food inflation (annual growth rate), health spending ($ *per capita*), growth rate (annual total), GDP *per capita* ($) for 14 OECD countries are present.

**Table 1:** Descriptive statistics of the variables for 14 OECD countries (2020).

	Meat consumption (kg *per capita*)					Crop production (tones)					Population	Total crop production per 1 m people (tones)	Producer support (total ratio)	Producer protection (% of gross farm receipts)	Household spending	Food inflation annual growth rate	GDP annual growth rate	Health spending	GDP *per capita*, ($)
	Beef and veal	Pork	Poultry	Sheep	Total	Wheat	Maize	Rice	Soybean	Total									
Australia	19.44	20.21	43.79	5.87	89.32	31164.50	200.79	380.00	44.06	31789.35	25,693,267	1237.25	3.29	1.00	50.66	9.32	−2.21	5627.32	55996.38
Canada	17.58	15.88	35.53	0.97	69.96	35188.18	13563.41	0.00	6358.55	55110.14	38,037,204	1448.85	8.18	1.05	57.16	2.40	−5.23	5828.32	46572.14
Chile	20.26	24.76	37.07	0.48	82.58	1300.00	594.48	117.57	0.00	2012.05	1,945,831	1034.03	2.43	1.00	57.77	6.74	−6.21	2412.75	24647.94
Colombia	8.62	8.99	31.70	0.14	49.46	5.00	1430.00	2005.82	75.00	3515.82	50,911,747	69.06	10.16	1.10	70.51	5.55	−7.05	1335.88	15306.34
Israel	23.21	1.31	64.44	1.53	90.49	85.00	81.00	0.00	0.00	166.00	9,215,113	18.01	15.36	1.16	48.42	0.0	−1.78	3057.41	40090.87
Japan	7.64	16.13	17.73	0.16	41.67	1037.00	0.00	7396.39	241.65	8675.04	126,146,099	68.77	43.50	1.66	53.79	1.20	−4.62	4665.64	42285.33
Korea	11.85	31.57	18.74	0.33	62.49	27.15	73.58	3996.48	98.98	4196.20	51,836,239	80.95	47.91	1.73	46.39	4.43	−0.71	3582.31	45403.24
Mexico	8.97	14.42	30.59	0.54	54.52	2957.48	275599.39	358.23	268.41	279183.51	127,792,286	2184.66	9.52	1.06	63.07	6.62	−8.06	1226.74	19106.97
New Zealand	11.55	19.06	40.08	3.45	74.14	482.06	181.77	0.00	0.00	663.83	50,902	13041.39	0.98	1.01	57.50	3.18	−1.06	4469.38	44698.27
Norway	12.81	21.10	17.84	4.48	56.22	468.97	0.00	0.00	0.00	468.97	5,379,472	87.18	53.40	1.61	43.98	3.26	−0.72	6536.09	62650.38
Switzerland	13.11	22.46	14.77	1.15	51.48	445.99	146.69	0.27	5.93	598.88	863,817	693.28	52.65	1.60	51.61	0.09	−2.51	7178.56	70555.78
Turkey	9.63	0.00	19.18	4.23	33.05	20500.00	6031.00	588.00	140.00	27259.00	83,384,688	326.91	26.03	1.16	56.77	13.85	1.83	1304.71	27554.34
United Kingdom	11.33	15.82	30.45	3.93	61.52	10132.99	0.00	0.00	0.00	10132.99	67,081,234	151.06	18.92	1.05	59.86	0.70	−11.03	5018.70	45644.39
United States	26.20	23.98	50.96	0.43	101.57	49696.42	360251.16	6738.04	112538.16	529223.78	33,150,108	15964.47	11.62	1.02	67.03	3.51	−2.77	11859.18	63480.86

The Bartlett test has given a significant result. Hence, the number of observations accepted as sufficient. We determined that a significant single factor explained 66.27% of the variance. In the loads explaining the factor, the highest load was carried out by GDP *per capita*, while the least was the GDP growth rate. As a result of the factor analysis with the regressional score method, we created the variable, Factor_1 (Table 2).

**Table 2:** Factor analysis of GDP annual growth rate, GDP *per capita* ($), and Health Spending *per capita* ($) variables.

	Explanatory of factor_1					
	Initial eigenvalues			Factor loadings		
	Total	% of Variance	Cumulative %	Variables	Component matrix Load	Coefficient
1.	1.988	66.266	66.266	GDP Growth rate	0.478	0.240
2.	0.900	30.002	96.268	Health Spending	0.910	0.458
3.	0.112	3.732	100.00	GDP *Per Capita*	0.965	0.485

Bartlett test *χ*^2^:17.954; *df*:3; *p* = 0,000450; *n* = 14.

**Figure 2:**
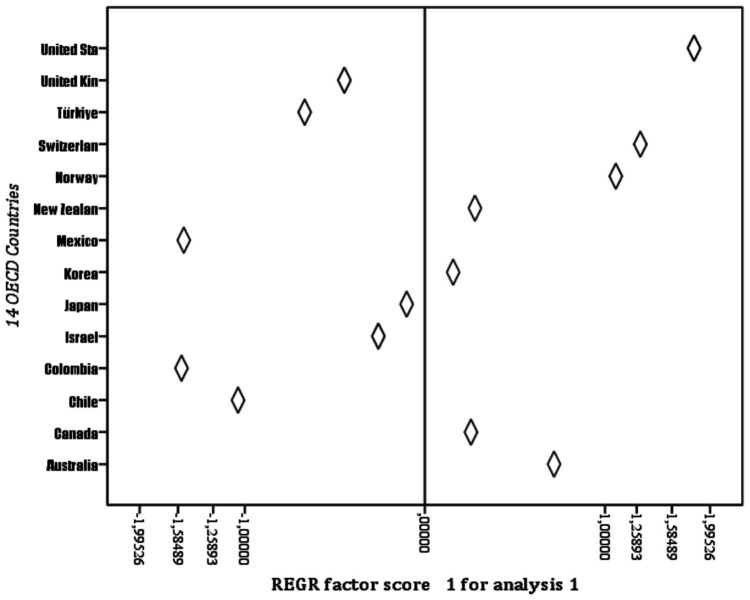
Logarithmic distribution of the observation values for the Factor_1 variable.

When the observation values for the Factor_1 variable are ranked (Figure 2), the highest country is the United States, and the lowest is Colombia (Table 3).

**Table 3:** Distances of variables according to Factor 1 and priority transformation.

Variables	Dimension 1	Dimension 2	Variables	Distances to Factor_1
Meat	–0.011	0.535		
Crop	1.351	–0.220	Meat	0.897
Support	–0.185	0.027	Crop	1.769
Protection	–0.408	–0.250	Support	0.373
Household	0.056	0.367	Protection	0.018
Food inflation	–0.386	–0.194	Household	0.790
Factor_1	–0.417	–0.266	Food inflation	0.078

The data priorities were created with a single matrix source using the MDS PROXSCAL. We used the Euclidean distance for the measurement, provided the *z*-score standardization through the transformation, and explained the proportional model. Since the standard deviation for crop production is very high, according to the OECD 2020 data, the countries were divided by their population and multiplied by 1 million to determine the total crop production obtained from 4 different crop production data per 1 million people.


$$\mathrm{Euclidean Distance}\;\left(x,y\right)=\sqrt{\overset{n=14}{\underset{i=1}{\sum }}{\left(x_{i}-y_{i}\right)}^{2}}\text{.}$$

**Figure 3:**
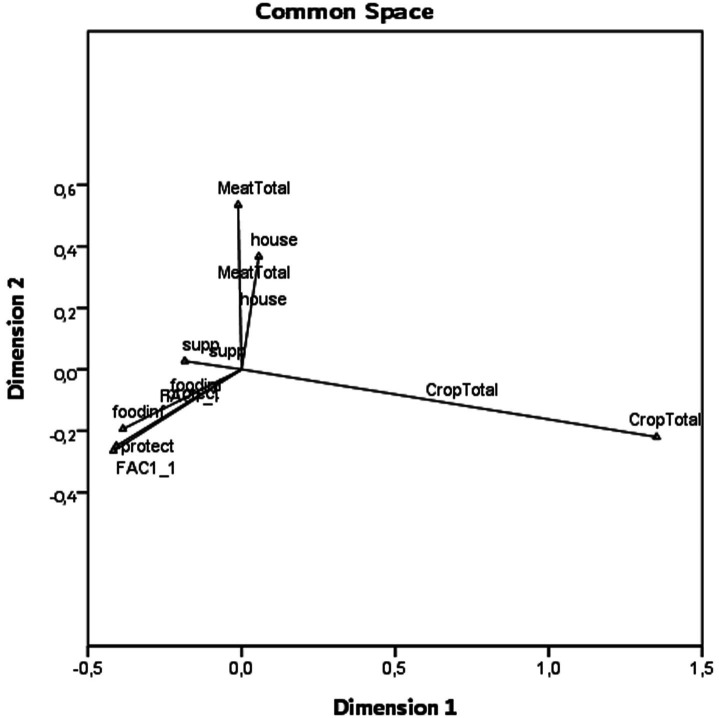
Variables in common space and Euclidean distances.

We explained the model under the Euclidean distance method (S-Stress: 0.00269). When we examined the variables in the space, it is seen that the total of the elements that make up the crop production is located at the farthest distance from Factor_1. The closest distance is occupied by producer protection (Figure 3). The distances are given in Table 3.

When we examined the distances of the variables according to Factor_1, we determined that the closest to the farthest is producer protection (0.018); food inflation (0.078), producer support (0.373), household spending (0.790), meat consumption (0.897), crop production (1.769; Table 3).

To determine the weights of the criteria, the ratio of each distance value to the sum of the distance values was calculated to create a proportional matrix. Then, the determined values were subtracted from 1 to rank the criteria from most successful to least successful, and the criteria weights were determined by calculating the ratio again (Table 4).

**Table 4:** Criteria weights matrix.

	Distances to Factor_1	Ratio matrix (x_i_)	Fixed (1–x_i_)	Criteria weights (W_i_)
Meat	0.897	0.228552737	0.771447263	0.154289453
Crop	1.769	0.450521561	0.549478439	0.109895688
Support	0.373	0.095106865	0.904893135	0.180978627
Protection	0.018	0.004690236	0.995309764	0.199061953
Household	0.790	0.201268933	0.798731067	0.159746213
Food inflation	0.078	0.019859668	0.980140332	0.196028066

We found the normalized decision matrix by normalizing the values in rows (i) and columns (j) for each observation (d) in the unweighted matrix (Tables 5, 6).


$$\frac{d_{ij}}{\sqrt{\sum_{k=1}^{14}d_{11}+\ldots +d_{146}}}\text{.}$$

After normalization, we determined the normalized matrix and found the values from before the weighted matrix (Table 7). To find the vector (V), we multiplied the normalized values for each observation by the weights:

After the determination of the vector, the positive benefit criterion values (A^*^) and negative cost criterion values (*A*^−^) for each column were calculated through the maximum and minimum column values. Then, the positive ideal solution distances (S^*^) and negative ideal solution distances (*S*^−^) were found, and the analysis was completed by determining the Closeness Coefficient (C^*^) with the formula.


$$J=\left[\;j=\left(1,2,3,4,5,6\right)\right];J:\mathrm{benefit}\text{,}$$

$$J^{\prime }=\left[\;j=\left(1,2,3,4,5,6\right)\right];J^{\prime }:\mathrm{cost}\text{,}$$

$$A^{*}=\left\{\left(\max_{i}v_{ij}\;|\;j\in J\right),\left(\min_{i}v_{ij}\;|\;j\in J^{\prime }\right)\right\}\text{,}$$

$$A^{-}=\left\{\left(\min_{i}v_{ij}\;|\;j\in J\right),\left(\max_{i}v_{ij}\;|\;j\in J^{\prime }\right)\right\}\text{,}$$

$$S\begin{array}{c} {}*\\ i \end{array}=\sqrt{\overset{14}{\underset{j=1}{\sum }}{\left(v_{ij}-v_{j}^{*}\right)}^{2}};i=1,2,3,4,5,6\text{,}$$

$$S\begin{array}{c} -\\ i \end{array}=\sqrt{\overset{14}{\underset{j=1}{\sum }}{\left(v_{ij}-v_{j}^{-}\right)}^{2};i=1,2,3,4,5,6}\text{,}$$

$$C\begin{array}{c} {}*\\ i \end{array}=\frac{S\begin{array}{c} -\\ i \end{array}}{S\begin{array}{c} -\\ i \end{array}+S\begin{array}{c} {}*\\ i \end{array}}\text{.}$$

We used the weighted matrix to calculate the benefit values (A^*^) and cost values (*A*^−^). We determined that the country with the highest proximity coefficient in the solution matrix was the most successful in terms of the criteria and weights in the comparative analysis. We also ranked the countries by their success.

**Table 5:** Unweighted matrix and criteria weights.

*Criteria:*	Meat	Crop	Support	Protection	Household	Food inflation
*Weight:*	*W_1_: 0.154289*	*W_2_: 0.109896*	*W_3_: 0.180979*	*W_4_: 0.199062*	*W_5_: 0.159746*	*W_6_: 0.196028*
*Alternatives*
Colombia	49.46	1237.25	10.16	1.1	70.51	5.55
Mexico	54.52	1448.85	9.52	1.06	63.07	6.62
Chile	82.58	1034.03	2.43	1	57.77	6.74
Turkey	33.05	69.06	26.03	1.16	56.77	13.85
United Kingdom	61.52	18.01	18.92	1.05	59.86	0.7
Israel	90.49	68.77	15.36	1.16	48.42	0
Japan	41.67	80.95	43.5	1.66	53.79	1.2
Korea	62.49	2184.66	47.91	1.73	46.39	4.43
Canada	69.96	13041.39	8.18	1.05	57.16	2.4
New Zealand	74.14	87.18	0.98	1.01	57.5	3.18
Australia	89.32	693.28	3.29	1	50.66	9.32
Norway	56.22	326.91	53.4	1.61	43.98	3.26
Switzerland	51.48	151.06	52.65	1.6	51.61	0.09
United States	101.57	15964.47	11.62	1.02	67.03	3.51

**Table 6:** Normalized matrix result.

	Meat	Crop	Support	Protection	Household	Food inflation
	*(r_i1_)*	*(r_i2_)*	*(r_i3_)*	*(r_i4_)*	*(r_i5_)*	*(r_i6_)*
*Alternatives*
Colombia (r_1j_)	*9.562718615*	*0.228657558*	*0.962745283*	*0.256860848*	*23.51156693*	*1.43754576*
Mexico(r_2j_)	*11.61943062*	*228.8232381*	*0.84527472*	*0.23851971*	*18.81160307*	*2.045274909*
Chile(r_3j_)	*26.65771434*	*51.26237119*	*0.055072721*	*0.212281693*	*15.78283003*	*2.120095893*
Turkey (r_4j_)	*4.269884036*	*5.123760535*	*6.319348694*	*0.285646246*	*15.24115686*	*8.95231301*
United Kingdom (r_5j_)	*14.79468784*	*1.094035397*	*3.338614289*	*0.234040566*	*16.94546807*	*0.02286819*
Israel (r_6j_)	*32.00916382*	*0.015551054*	*2.200424212*	*0.285646246*	*11.08739997*	*0.000000*
Japan (r_7j_)	*6.787661151*	*0.226741211*	*17.64828456*	*0.584963432*	*13.68306005*	*0.067204477*
Korea (r_8j_)	*15.26490838*	*0.314171018*	*21.4080113*	*0.635337878*	*1.1772136*	*0.915889677*
Canada (r_9j_)	*19.13254548*	*10.5281134*	*0.624066099*	*0.234040566*	*15.45128434*	*0.268817907*
New Zealand (r_10j_)	*21.48712528*	*8154.177825*	*0.00895728*	*0.216548555*	*15.63564617*	*0.471943438*
Australia (r_11j_)	*31.18678322*	*73.39176599*	*0.100952198*	*0.212281693*	*12.13697662*	*4.053848707*
Norway (r_12j_)	*12.35534382*	*0.364389742*	*26.59539823*	*0.550255375*	*9.147250962*	*0.495987706*
Switzerland (r_13j_)	*1.35977276*	*23.04356967*	*25.85358271*	*0.543441133*	*12.59644117*	*0.000378025*
USA (r_14j_)	*4.32775608*	*12219.16202*	*1.259320403*	*0.220857873*	*21.24802571*	*0.574976301*

**Table 7:** Weighted normalized decision matrix with benefit (A^*^) matrix and cost (A^−^) matrix.

	Weighted normalized decision matrix					
	Meat^*^	Crop^*^	Support^*^	Protection^*^	Household^−^	Food inflation^−^
Colombia	1.475426624	0.02512848	0.17423632	0.051131222	3.755883779	0.281799315
Mexico	1.792755594	25.14668719	0.152976658	0.047480199	3.005082351	0.400931285
Chile	4.113004163	5.633513551	0.009966985	0.042257208	2.521247328	0.415598298
Turkey	0.658798072	0.563079189	1.14366705	0.0568613	2.43471709	1.754904606
United Kingdom	2.282664294	0.120229773	0.60421783	0.046588572	2.706974351	0.004482807
Israel	4.938676376	0.001708994	0.398229753	0.0568613	1.771170157	0.00000000
Japan	1.047264526	0.024917881	3.193962308	0.116443963	2.185817025	0.013173964
Korea	2.355214364	0.03452604	3.874392492	0.126471599	1.625771331	0.179540082
Canada	2.951949976	11.04760618	0.112942626	0.046588572	2.468284159	0.052695854
New Zealand	3.315236807	896.1089821	0.001621076	0.043106578	2.497735263	0.092514159
Australia	4.811791724	8.065438617	0.01827019	0.042257208	1.938836052	0.794668122
Norway	1.906299239	0.040044861	4.813198657	0.10953491	1.461238701	0.097227511
Switzerland	1.598403672	2.532388943	4.678945901	0.108178453	2.012233773	7.41035E-05
United States	6.222147426	1342.833217	0.227910078	0.0439644	3.394291641	0.112711492
*A* ^*^	6.222147	1342.833217	4.813199	0.126472	1.461239	0.000000
*A* ^−^	0.658798	0.001709	0.001621	0.042257	3.755884	1.754905

As a result of the analysis, the United States was determined to have the lowest risk in terms of food insecurity. New Zealand was in second place, and Mexico was in third. Colombia was found to be the country with the highest risk of food insecurity (Table 8).

**Table 8:** Rankings of 14 OECD countries from least to most food insecurity risk (2020).

Rank	Countries	PIS score	NIS score	Closeness coefficient
1	United States	4,978,059,496	1,342,844,104	0.99630659
2	New Zealand	446,7,608,229	896,1,136,357	0.66731006
3	Mexico	1,317,703,183	25,21,855,805	0.018778874
4	Canada	1,331,798,304	11,48,208,296	0.008547793
5	Australia	1,334,77,746	9,300,273,407	0.006919446
6	Chile	1,337,21,048	6,853,240,146	0.005098895
7	Switzerland	134.308923	5,940,425,763	0.004412575
8	Norway	1,342,800,111	5,720,736,254	0.00424223
9	Israel	1,342,839,416	5,049123524	0.00374595
10	Korea	1,342,804,609	4,990,341,484	0.003702597
11	Japan	1,342,819,442	3,980,794,681	0.002955742
12	United Kingdom	1,342,725,943	2,679,238,278	0.001991399
13	Turkey	1,342,288,184	1,834,420,945	0.001364772
14	Colombia	1,342,826,483	1,693,323,546	0.001259426

## 5. Discussion

Approximately 13 million children and 23 million adults in the United States lacked food security, a 2005 study indicated (39). In their study, Coleman-Jensen et al. (40) stated approximately 41 million Americans were at risk of food insecurity in 2016. Although there appears to be little difference when considering population growth in recent years, the high healthcare expenditure *per capita* and GDP *per capita* in the United States suggest that the threat of food insecurity may be minimal. However, it is important to note that the increasing healthcare expenditures and global policies prioritizing protective healthcare services require a reevaluation of food insecurity and its dimensions. In this context, the consumption of domestic agricultural production and meat consumption should also be considered. Unpredictable political developments such as wars, conflicts, and embargoes increase the risk of dependence on external sources for essential needs (41). For example, we have witnessed how the tension between Russia and several other countries in 2022 and Europe’s need for energy have led to changes in these countries’ strategies. The United States is relatively less at risk due to its production and export capacity. These reasons also apply to Canada based on recent data. Despite its small population, New Zealand is a significant agricultural producer and primarily uses its land for pastoral farming (42). Therefore, New Zealand can reduce the risk of food insecurity through its production capabilities, and they rank second in our study. On the other hand, South Korea, despite its relatively high GDP *per capita*, does not seem to have a successful ranking in terms of food insecurity. A study including 10,655 Koreans suggests 4,988 (46.8%) were mildly insecure and 299 (2.8%) were moderately/severely insecure (43). South Korea also does not rank among the countries with high agricultural and meat production. The same is true for Israel, which has a similar proximity score to South Korea. Efrati Philip et al. (44) noted that the prevalence of non-communicable diseases, obesity, and subjective poor health is significantly high among the general Israeli population, also indicating that users of food pantries are at risk of food insecurity. In the case of Mexico, agriculture-based production plays a significant role. While its GDP *per capita* and healthcare expenditures are not in a favorable position, its meat consumption is relatively high compared to other countries. These situations provide two indicators: the ability to produce and consume agricultural products and meat, or the capacity to purchase them, reduces the risk of food insecurity. The same situation applies to Chile, which is known for its high capacity for milk and meat production (45). A 2007 study in Colombia found that child food insecurity was significantly related to being underweight and mentioned the high prevalence of food insecurity in Bogota (46). Upon closer examination, Colombia does not have high scores regarding healthcare expenditure, GDP, agricultural production, or meat consumption. Therefore, they rank last in our study. In their research, Cuesta and Castro-Rios (47) mentioned the iron, vitamin A, and zinc deficiencies, low availability of food, quality and safety issues of food, and poor eating habits among individuals living in Colombia, and they suggested incorporating mushrooms into the food culture. We can interpret these results obtained with different dimensions as being related to our macro-level comparison analysis. In their study, Borelli et al. (48) mentioned that Turkey’s risk of food insecurity, which is also influenced by housing, has been increasing in recent years. In a 2017 study in Turkey, Ipek (49) found that an increase in income level, education level, and healthcare expenditures significantly reduced food insecurity. In developing countries, the increase in healthcare expenditure share of income and the structural breaks in income are expected to have higher marginal benefit outputs (50). For example, in the case of the United States, Popescu’s (51) study did not yield significant marginal benefit results. In our study, both Switzerland and Norway were in the middle ranks. This is because both countries have average levels of crop production and meat consumption but are among the top ranks in terms of GDP *per capita* and healthcare expenditures. Therefore, their proximity coefficients have yielded similar results. While in Japan, there is low meat consumption, United Kingdom’s producer support is weaker and crop production per million people is low, and both countries’ economies are shrinking. In the United Kingdom, there is increasing evidence of the use of food banks, and voices are rising about the potential link to long-term poverty, austerity, precarious employment, the rising cost of living, low wages, and cuts to social assistance and public services. (51). Despite providing food assistance to 1.6 million people living in the United Kingdom each year, the Trussell Trust—the United Kingdom’s largest foodbank network—reports that food insecurity is much more widespread in the United Kingdom (52). All of this has contributed to Japan and the United Kingdom being ranked lower on the list despite their high healthcare spending and GDP *per capita*. Studies in the literature have shown that nearly every country has citizens who are at risk of food insecurity, and solutions to reduce this risk are being sought (52, 53). Therefore, our study is important in terms of comparative macro situational assessment, rather than focusing on solutions for a single country. Although it is often repeated that the global healthcare system is moving towards proactive solutions that protect health before illness arises, rather than reactive solutions, perhaps the first step in the solution will be to reduce the risk of food insecurity. If indicators such as food aid, nutrient balance, and food waste are expanded in the literature to cover countries, research on the increasing trend of food insecurity risk will increase.

## 6. Conclusion

Food insecurity is a global issue affecting millions of individuals and families. While there are varying levels of risk in different countries, our study highlights the importance of considering multiple dimensions such as healthcare expenditure, GDP, agricultural production, and meat consumption. The ability to produce and consume agricultural products and meat, or the capacity to purchase them, reduces the risk of food insecurity. Our study provides a comparative macro situational assessment, highlighting the need for solutions to reduce this risk on a global scale. As the healthcare system moves towards proactive solutions that protect health before illness arises, reducing the risk of food insecurity may be an important first step. Further research on the increasing trend of food insecurity risk and expanded indicators such as food aid, nutrient balance, and food waste can help in finding solutions to this global issue.

Short-term, medium-term, and long-term policy recommendations can be made for countries based on the results of the study. Short-term recommendations may include innovations in food safety legislation, tightening of inspections, encouragement of obtaining documents related to food safety standards, organizing public awareness campaigns, and adding food safety to education curricula. Medium-term recommendations may include developing a national strategic plan and utilizing sustainable agricultural practices and support for producers. Longer-term recommendations may include the promotion of research and development activities for food safety, encouragement of the agricultural sector and food industry to sustainably produce in an ecologically balanced manner, imposition of mandatory continuing education for food producers and businesses, the establishment of international cooperation and certification systems, and deepening of research on international production and distribution of agriculture and animal husbandry. Additionally, it should not be forgotten that practices that generally reduce income inequality in countries are extremely important for food insecurity.

Although some limitations, the study’s novel modeling approach has produced results consistent with previous research, indicating the robustness of these findings. We believe that our study provides a valuable baseline for future research, and that future studies can build upon our work by exploring different variables and larger datasets over time. Comparative studies could be particularly useful in this regard.

## Data availability statement

The datasets presented in this study can be found in online repositories. The names of the repository/repositories and accession number(s) can be found in the article/supplementary material.

## Author contributions

SY and AG contributed to the idea, conception, and design of the study. SY organized the database, performed the statistical analysis, and wrote the background and discussion. AG wrote the first draft of the manuscript and the abstract and introduction. All authors contributed to the article and approved the submitted version.

## Conflict of interest

The authors declare that the research was conducted in the absence of any commercial or financial relationships that could be construed as a potential conflict of interest.

## Publisher’s note

All claims expressed in this article are solely those of the authors and do not necessarily represent those of their affiliated organizations, or those of the publisher, the editors and the reviewers. Any product that may be evaluated in this article, or claim that may be made by its manufacturer, is not guaranteed or endorsed by the publisher.
